# Reviewing the development, evidence base, and application of the revised dengue case classification

**DOI:** 10.1179/2047773212Y.0000000017

**Published:** 2012-05

**Authors:** O Horstick, J Farrar, L Lum, E Martinez, J L San Martin, J Ehrenberg, R Velayudhan, A Kroeger

**Affiliations:** 1 Special Programme for Research and Training in Tropical Diseases (TDR_WHO), Geneva, Switzerland; 2 Institute of Public Health, University of Heidelberg, Germany; 3 Oxford University Clinical Research Unit, Ho Chi Minh City, Vietnam; 4 Department of Paediatrics, Faculty of Medicine, University of Malaya, Kuala Lumpur, Malaysia; 5 Instituto de Medicina Tropical Pedro Kouri, Marianao, Ciudad de la Habana, Cuba; 6 Panamerican Health Organization, World Health Organization, Regional Office for the Americas, Panama City, Panama; 7 World Health Organization, Regional Office for the Western Pacific Region, Manila, The Philippines; 8 World Health Organization, Department of Control of Neglected Tropical Diseases (NTD), Geneva, Switzerland; 9 Liverpool School of Tropical Medicine, UK

**Keywords:** Dengue, Case classification, Evidence-based approaches

## Abstract

With the example of dengue, an evidence-based approach to prospectively develop a case classification is described, gathering evidence for identifying strength and weaknesses of the existing model, collecting new data describing the disease as it occurs globally, further developing a new model that can be applied in practice and field testing the newly developed model in comparison to the previous model. For each step in this process, the highest available level of evidence has been applied. This process has been initiated by the World Health Organization’s (WHO) Special Programme for Research and Training in Tropical Diseases (TDR) and WHO’s Department for Control of Neglected Tropical Diseases (NTD), developing the following for dengue. Since the early 1970s, dengue has been classified into dengue fever, dengue haemorrhagic fever grades I and II and dengue shock syndrome grades III and IV (DF/DHF/DSS). However, in recent years, a growing number of dengue clinicians have questioned the shortcomings of this scheme. The issues have revolved around the complexity of confirming DHF in clinical practice, misclassifying severe cases as DF, and the emphasis on haemorrhage rather than plasma leakage as the underlying problem in most severe dengue cases. Step 1: A systematic literature review highlighted the shortcomings of the DF/DHF/DSS scheme: (1) difficulties in applying the criteria for DHF/DSS; (2) the tourniquet test has a low sensitivity for distinguishing between DHF and DF; and (3) most DHF criteria had a large variability in frequency of occurrence. Step 2: An analysis of regional and national dengue guidelines and their application in the clinical practice showed a need to re-evaluate and standardize guidelines as the actual ones showed a large variation of definitions, an inconsistent application by medical staff, and a lack of diagnostic facilities necessary for the DHF diagnosis in frontline services. Step 3: A prospective cohort study in seven countries, confirmed the difficulties in applying the DF/DHF/DSS criteria even in tertiary care hospitals, that DF/DHF/DSS do not represent levels of disease severity and that a clear distinction between severe dengue (defined by plasma leakage and/or severe haemorrhage, and/or organ failure) and (non-severe) dengue can be made using highly sensitive and specific criteria. In contrast, the sub-grouping of (non-severe) dengue into two further severity levels was only possible with criteria that gave approximately 70% sensitivity and specificity. Step 4: Three regional expert consensus groups in the Americas and Asia concluded that ‘dengue is one disease entity with different clinical presentations and often with unpredictable clinical evolution and outcome’ and that, revising the results of Step 3, DF/DHF/DSS is not related to disease severity. Step 5: In a global expert consensus meeting at WHO in Geneva/Switzerland the evidence collected in Steps 1–4 was reviewed and a revised scheme was developed and accepted, distinguishing: dengue with or without warning signs and severe dengue; the further field testing and acquisition of further prospective evidence of the revised scheme was recommended. Step 6: In 18 countries, the usefulness and applicability of the revised classification compared to the DF/DHF/DSS scheme were tested showing clear results in favour of the revised classification. Step 7: Studies are under way on the predictive value of warning signs for severe dengue and on criteria for the clinical diagnosis of dengue which will complete the evidence foundation of the revised classification. The analysis has shown that the revised dengue case classification is better able to standardize clinical management, raise awareness about unnecessary interventions, match patient categories with specific treatment instructions, and make the key messages of patient management understandable for all health care staff dealing with dengue patients. Furthermore, the evidence-based approach to develop prospectively the dengue case classification could be a model approach for other disease classifications.

## Background and justification of the review

**Figure 1:**
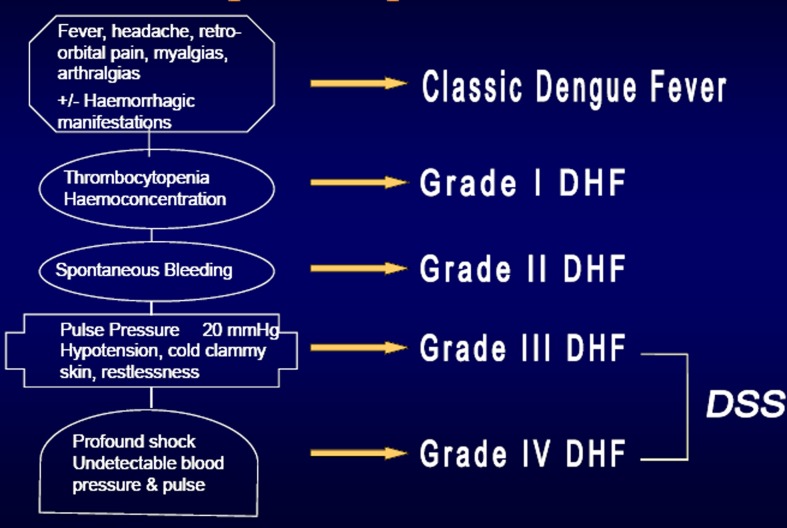
The categories of the 1975 and 1997 WHO dengue case classification.

Since the early 1970s, dengue has been classified into dengue fever, dengue haemorrhagic fever, and dengue shock syndrome (DF/DHF/DSS) as described in the WHO guidelines for dengue from 1975 and 1997.^1^,^2^ The core element was the precise definition of DHF with well-described criteria (Fig. 1). Later on a severity grading was added, dividing DHF into four levels of disease severity, grades I–IV. Grades III and IV represent DSS giving five different categories of disease. Numerous publications underline the strength of the DF/DHF/DSS classification and its merits in clinical case management. Certainly, the classification had it merits describing retrospectively patterns of disease, as these occurred in the early days of the dengue pandemic. However, in recent years, a growing number of clinicians and authors pointed to deficiencies in the DF/DHF/DSS classification and its application.

Shortcomings were reported in the areas of clinical management, surveillance, and research with a set of studies published in the 1990s and early 2000s. Examples are Rigau-Perez:^3^ ‘The WHO dengue haemorrhagic fever definition needs to be simplified, standardized, and tested’. Deen *et al.*:^4^ ‘A large multicentre descriptive study is needed to obtain the evidence to establish a robust dengue classification scheme for use by clinicians, epidemiologists, public-health authorities, vaccine specialists, and scientists involved in dengue pathogenesis research’ and Halstead:^5^ ‘It has proved difficult to satisfy the WHO case definition of hemoconcentration in several settings’. The latter article clearly describes the dilemma in the practical use of the model DF/DHF/DSS: ‘Clinicians encountered problems in establishing a diagnosis of hemoconcentration during the acute illness phase and epidemiologists encountered the problem of making a diagnosis retrospectively’.

In practice, case classifications serve multiple purposes. They can guide clinicians in diagnosing a particular condition, deciding on the level of severity and determining the treatment; case classifications are also needed in epidemiological surveillance, for routine data collection, for outbreak investigations and when testing the efficacy of vaccines or drugs for outcome measurement. It was assumed and frequently quoted in the literature that DF represents the mild form of dengue, DHF the severe form, and DSS the very severe and life-threatening form. However, both clinicians and epidemiologists noticed that DF could be a fatal illness; DHF often causes mild illness only and more often, that when using the rigid DHF criteria patients could not be classified at all. As the disease spread globally and moved beyond an overwhelmingly paediatric disease, these limitations became more apparent. These shifts in the pattern of disease led to calls for the development of an evidenced based DF/DHF/DSS classification scheme developed on prospectively gathered data worldwide.

This process was initiated by the World Health Organization’s (WHO) Special Programme for Research and Training in Tropical Diseases (TDR) and WHO’s Department for Control of Neglected Tropical Diseases (NTD) in August 2003 when during the biannual International Dengue Course in La Habana/Cuba dengue clinicians and epidemiologists from all over the world discussed the utility of the DF/DHF/DSS classification in clinical practice, disease surveillance, and research.

The purpose of this paper is to describe and review — as a potential model for other disease classifications — the step-by-step approach which was used for approaching a revised dengue case classification based whenever possible on prospectively gathered evidence.

## Underlying Methods for Each Step of Development of the Revised Case Classification

The methods chosen for this step-by-step process are a mix of quantitative and qualitative methods; this applies to the overall process for developing the revised dengue case classification, but also for the individual studies. In summary, the following methods have been used:

- clinical research with a prospective cohort study;
- comparative analyses of existing data;
- systematic literature reviews;
- applying and testing the above mentioned research in real-life settings using questionnaire interviews, in depth interviews with key informants and focus group discussions (FGDs);
- expert consensus groups analysing the process and reviewing the evidence, but also generating further ideas on how to proceed.

Data quality has been ensured in each study according to the study type, e.g. for studies collecting primary data strict data monitoring, double data entry, central data management, and analysis; for the systematic reviews following quality criteria for systematic reviews, including double data reviewing were applied.

The individual studies discussed have applied the following methods:

### Step 1: Systematic review of the benefits and shortcomings of the DF/DHF/DSS classification^6^

‘Classifying dengue: a review of the difficulties in using the WHO case classification for dengue haemorrhagic fever’.

This study is a systematic review of studies analysing the use of the model DF/DHF/DSS, with two independent reviewers, including all English-language publications from 1975 (when the DF/DHF/DSS model was developed) until the publication date.

### Step 2: Review of the use of dengue guidelines and application of the DF/DHF/DSS classification^7^

‘Comparison and critical appraisal of dengue clinical guidelines and their use in Asia and Latin America’.

This study is a comparative document analysis of 13 dengue guidelines and triangulating the information on their use and applicability with questionnaires and FGDs with 858 health care providers in seven countries in Asia and Latin America.

### Step 3: Prospective multi-centre clinical cohort study^8^

‘Evidence for a revised dengue case classification: a multi-centre prospective study across Southeast Asia and Latin America’.

This study is a prospective clinical cohort study in seven countries, describing the clinical and laboratory profile of paediatric and adult dengue cases. Cases were followed up daily with detailed case report forms under standardized operating procedures, and subsequently categorized into one of three intervention groups according to the overall level of medical and nursing support required. Using an *a priori* analysis plan, the clinical and laboratory profiles characteristic of these intervention categories were explored to develop a revised case classification based on disease severity.

### Step 4: Expert consensus meetings evolving in the context of the multi-centre study

Three expert consensus meetings on the use of the dengue case classification DF/DHS/DSS: (1) La Habana/Cuba and (2) Kuala Lumpur/Malaysia (both 2007) and in (3) Heidelberg/Germany (2008).

Two regional expert consensus meetings were held determining the requirements for a revised dengue case classification and reviewing the progress made in the DENCO study (see Step 3) in La Habana/Cuba and Kuala Lumpur/Malaysia 2007, with dengue experts from different professional backgrounds, and from different countries in Asia and Latin America and the Caribbean, respecting country, regional, and gender representation.

After reviewing the available evidence, consensus was sought in plenary discussions, break-out discussion groups, presentation of group results, and final agreement in plenary discussions. Disagreements that could not be solved by consensus have been documented as such. Additionally, a global expert consensus meeting was held in Heidelberg/Germany in 2008, summarizing the two regional meetings and applying the same methodology as described above.

### Step 5: Global expert consensus meeting for the joint analysis of the multi-centre study findings and developing the revised case classification

A global expert consensus meeting on the revised dengue case classification in Geneva/Switzerland 2008, with dengue experts from different professional backgrounds, respecting country, regional, and gender representation. Consensus was reached in a similar process as described under Step 4. Additionally, each participant was asked individually at the end of discussions if he/she would agree to the conclusions. The meeting was followed by a smaller editorial committee meeting for the forthcoming global dengue guidelines.^9^

### Step 6: Study to test the user-friendliness and applicability of the revised dengue classification in 18 countries (‘validation study’)^10^

‘Usefulness and applicability of the revised dengue case classification by disease severity: multi-center study in 18 countries’.

This multi-centre study was conducted to test the usefulness and applicability of the revised dengue case classification, using prospective and retrospective reviews of medical charts, semi-structured staff interviews, and focus group discussions.

### Step 7: Further analysis of the predictive value of warning signs for severe dengue

The study was prepared in 2009 and started in 2011 with the objective to collect prospective data for testing the predictive value of clinical and laboratory warning signs for severe dengue in 15 countries using the following tools: prospective clinical data using a standardized case report form and staff questionnaires.

## Reviewing the Results of Each Step

The process to reach an evidence-based revised dengue case classification is presented according to the steps described in the above mentioned section.

### Step 1: Systematic review of the benefits and shortcomings of the DF/DHF/DSS classification

The systematic review^6^ identified 37 papers reporting on the use of the DF/DHF/DSS classification. Most studies stated that it was not possible to meet in addition to fever all three criteria of DHF (thrombocytopaenia, plasma leakage, and haemorrhagic manifestations often determined by the tourniquet test) so that alternative models to classify dengue were locally used. Specifically, the positive tourniquet test representing the minimum requirement of a haemorrhagic manifestation did not distinguish between DHF and DF. In cases of DHF, thrombocytopaenia was observed in a range of 8.6–96%, plasma leakage in 6–95%, and haemorrhagic manifestations in 22–93%. It was thought that the low sensitivity of the DHF criteria could be due to failure to repeat the tests or physical examinations at the appropriate time, early intravenous fluid therapy, and lack of adequate resources in an epidemic situation, and perhaps a considerable overlap of clinical manifestations of the different dengue entities. The study underlines the shortcomings of the DF/DHF/DSS model in clinical practice and particularly during dengue outbreaks.

### Step 2: Review of regional and national dengue guidelines and application of the DF/DHF/DSS classification

Santamaria *et al.*^7^ examined how guidelines and case classification are used in the reality of national health systems. In this study, 13 guidelines from Asia and Latin America were analysed and health care providers were interviewed using questionnaires and FGDs. It was found that there is a need to re-evaluate and standardize guidelines, since there were large differences and inconsistencies in their application: for example, in Latin America, only Brazil, Cuba, and Ecuador included the DF/DHF/DSS model in the national guidelines, whereas Colombia, El Salvador, and Mexico included different criteria. Brazil added a further category ‘complicated dengue’. Furthermore, the study identified a large number of staff incorrectly applying guidelines and particularly the case classification and also the unavailability of diagnostic tests at frontline services necessary to establish the DHF criteria. Furthermore, inaccessibility of guidelines was wide spread and a lack of training about how to use them was frequently mentioned.

### Step 3: Prospective multi-centre clinical cohort study

Primary data on dengue cases have subsequently been collected in the largest ever prospective cohort study in seven countries of Asia and Latin America, the Dengue Control (DENCO) study:^8^ patients above 6 months of age with clinically suspected dengue and fever for less than 7 days were enrolled. All patients were followed daily by trained study physicians using standardized case report forms describing clinical, laboratory, diagnostic, and management information in detail. All management decisions, including whether to admit to hospital, were made at the discretion of the treating clinicians following local policies. Haematocrit and platelet measurements were performed at least once daily, with full blood count, liver, and renal function tests done at least twice during the episode. Among hospitalized patients, a right lateral decubitus X-ray and/or ultrasound were carried out within 24 hours of defervescence. Two thousand two hundred and fifty-nine patients were recruited. A total of 230 (13%) of the 1734 laboratory confirmed patients required major interventions, with approximately 5% of patients progressing to this level of severity in hospital. Applying the DF/DHF/DSS criteria, 47/210 (22%) of patients with shock did not fulfil all criteria necessary for dengue haemorrhagic fever. Specific criteria of severe plasma leakage, severe bleeding, and severe organ involvement (details later) were able to identify dengue patients who required major clinical interventions; thus severe dengue and (non-severe) dengue could be distinguished with a 96% sensitivity and 100% specificity. In addition, warning signs for disease progression towards severe dengue could be identified (persistent abdominal pain and tenderness, lethargy, mucosal bleeding, and decreased platelet count); for the other warning sign candidates, the sample size was too small (see Step 7). All attempts to find highly sensitive indicators for a sub-classification of the (non-severe) dengue group failed and the best combination of diagnostic criteria did not reach 80% sensitivity and specificity.

### Step 4: Expert consensus meetings

Two expert consensus meetings on the evolving revised dengue case classification were organized in Asia (Kuala Lumpur/Malaysia; 68 participants) and Latin America/Caribbean (La Habana/Cuba, 42 participants) in 2007. One of the main conclusions was that ‘dengue is just one disease entity with different clinical presentations and often with unpredictable clinical evolution and outcome’. The participants of the two meetings also revised the evidence and concluded that ‘the current classification (DF, DHF, and DSS) is not clearly correlated with disease severity, therefore it is recommended to establish a validated dengue classification using levels of severity’. It was also recommended ‘to further develop treatment guidelines taking into account warning signs for severe disease’. These results have been further discussed and agreed on in a third expert consensus group in Heidelberg/Germany in 2008.

### Step 5: Global expert consensus meeting

**Figure 2:**
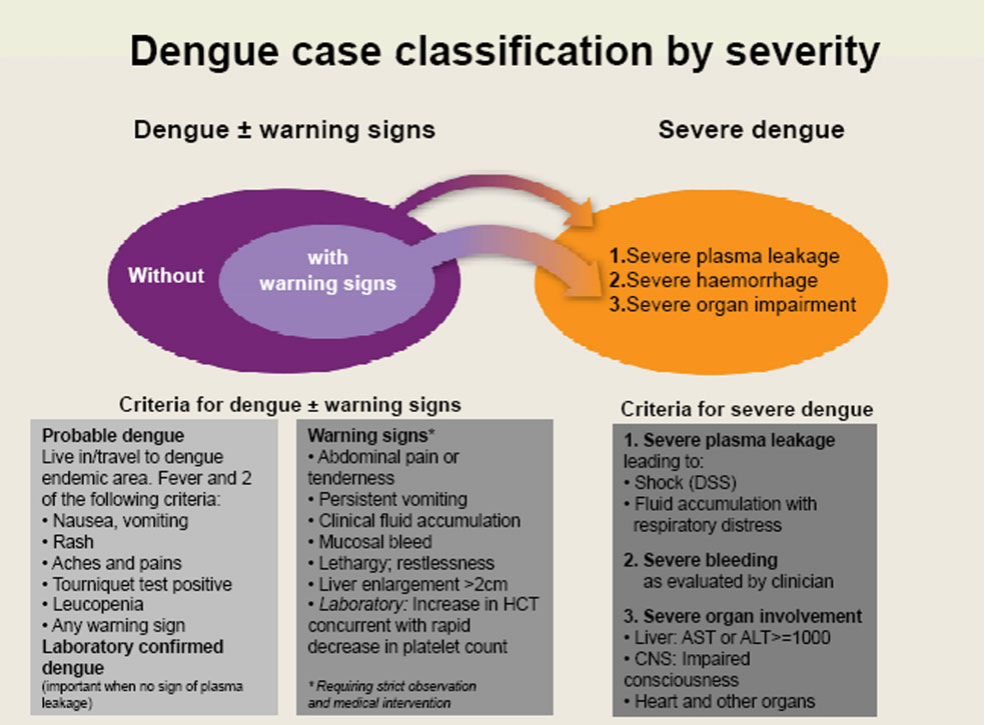
The revised dengue case classification by severity.

The evidence of the studies above and expert recommendations were reviewed in a global meeting on dengue case classification held at WHO in Geneva/Switzerland (September 2008). Sixty-eight clinical, epidemiological, virological, and immunological experts from all dengue endemic WHO Regions agreed that the evidence supports a binary classification system representing two clear entities, severe dengue, and dengue — with the notion that the term ‘non-severe dengue’ should be avoided as any dengue case can turn into a severe one. It was reiterated that any patient with dengue may progress to severe dengue, but it was also highlighted that patients who exhibit warning signs are at greater risk of disease progression and merit careful observation. Therefore the model ‘dengue +/− (i.e. with or without) warning signs’ and ‘severe dengue’ was recommended for the revised dengue case classification (Fig. 2). It was also recommended that the revised case classification should be field tested against the DF/DHF/DSS model.

### Step 6: Study to test the user-friendliness and applicability of the revised dengue classification in 18 countries (‘validation study’)

Barniol *et al.*,^10^ following the recommendation of the expert meeting in Geneva 2008, compared in 18 countries the revised case classification with the DF/DHF/DSS classification regarding user-friendliness, applicability in different clinical settings and in dengue surveillance, as well as its ability to distinguish at which level of care the individual patient had to be treated (triage). The study sites were selected based on geographic location, level of care provided, incidence of dengue in each site, and experience with dengue clinical case management. The sites represent the range of care offered in the local health care system from basic health centres to large teaching hospitals. As in many previous studies, it was reconfirmed that the applicability of the DF/DHF/DSS model was limited, even when strict DHF criteria were not applied; 13.7% of dengue cases could not be classified by experienced reviewers as compared to 1.6% who could not be classified with the revised classification; this was of particular concern, when severe dengue cases could not be classified in the DF/DHF/DSS system. Mismatches between the two systems were frequent, as the DF/DHF/DSS system is not a severity classification. The applicability of the revised classification to retrospective data sets — as in the case of dengue surveillance — was also favourable. Acceptance and perceived user-friendliness of the revised system were overwhelmingly high particularly in relation to triage and case management; however, the need for training, dissemination, and further research on the definition of ‘probable dengue and warning signs’ was underlined. It was concluded that the revised dengue classification has a high potential for facilitating dengue case management and surveillance. There was strong support for prospective studies designed to show the utility of the revised classification scheme in supporting clinical triage, clinical management, epidemiology, and other public health research. The aim of the revised scheme is to improve the outcome of patients with dengue and aid future research.

### Step 7: Further analysis of the predictive value of warning signs for severe dengue

A complementary study in 15 countries has been initiated to determine the predictive value of warnings signs for severe dengue other than the two already analysed in the DENCO study (Step 3). This has practical relevance for clinical management: Knowing the predictive value of defined signs and symptoms or combinations of them will help the clinician to decide when a dengue patient can be treated at home, in a general hospital unit, or under strict observation. The study findings will also be relevant for the endpoint measurement of drug or vaccine trials.

## Discussion of the Revised Case Classification

### Theory of disease classifications applied to the DSF/DHF/DSS model

Classifications should ideally fulfil the following criteria, as defined in the theory of epidemiology:^11^ ‘a) naturalness — the classes correspond to the nature of the thing being classified, b) exhaustiveness — every member of the group will fit into one (and only) class in the system, c) usefulness — the classification is practical, d) simplicity - the subclasses are not excessive and constructability, e) the set of classes can be constructed by a demonstrably systematic procedure’.

When examining the DF/DHF/DSS classification against the above mentioned criteria, shortcomings become evident:

1. Naturalness: DHF emphasizes haemorrhagic symptoms; however, there is a consensus that the risk of dengue is predominantly linked to plasma leakage, where the critical phase is determined by plasma leakage and not by haemorrhage.^12^ Therefore, the term ‘DHF’ does not ‘correspond to the nature of the thing being classified’. Furthermore, the results of the systematic review presented in Step 1, clearly show that there is a great variation of haemorrhagic symptoms unrelated to the overall severity of disease.
2. Exhaustiveness: The DF/DHF/DSS model does not classify ‘every member of the group’ with 20–30% of cases not being classified in the DF/DHF/DSS scheme as has been confirmed in Steps 1–3 and also in Step 6.
3. Usefulness: The model DF/DHF/DSS is limited, since criteria such as repeated measurements of platelet counts depend on the availability of laboratory equipment, leaving out many health centres in poor areas. This shortcoming has been especially mentioned in the expert consensus meetings described under Step 4.
4. Simplicity of the model is not the case, with five different categories (DF, DHFI, DHFII, DHFIII, and DHFIV, the latter two being called DSS) since many different alternative models have been developed worldwide.
5. ‘Based on a systematic procedure’: The DF/DHF/DSS model was not result of a ‘systematic procedure’ but evolved from the clinical experience at one centre in Asia which was at that time the best reflection of knowledge about the nature of the disease (WHO 1975 and 1997).^1^,^2^ It is not a surprise that when in 2009 the DF/DHF/DSS model was retrospectively tested against an existing dataset collected in a well-equipped tertiary level hospital in Thailand, 32% of cases requiring intervention were missed.^13^

### Introducing a revised case classification

A revision of a disease case classification is of course a major change, not only for health care professionals treating cases of dengue, but also for surveillance officers, researchers, health financing institutions, and last but not least the public. However, if the shortcomings of the model DF/DHF/DSS are as clear as it is described, and the model dengue +/− warning signs and severe dengue performs much better (as it emerges from the studies described above), a change towards the revised case classification is highly desirable.

Introducing an evidence-based revised case classification implies more than just producing primary research. The Bamako Call to Action on Research on Health asks specifically researchers to bridge the gap between research and action: ‘Knowledge translation was emphasized as an essential priority for governments: to link evidence to policy and to disseminate widely research results for maximum public use’.^14^ Systematic reviews and expert consensus rounds, involving policy makers, help to bridge the gap of knowledge translation.^15^ This approach combined with mixed research methodologies was applied in the process towards a revised dengue classification as described in this article.

Certainly, the step-by-step approach chosen in this process, always aiming at the highest available evidence for each step, represents a model approach for other disease classifications.

### Advantages and potential limitations of the revised dengue case classification

A systematic step-by-step procedure has been employed to achieve a revised dengue classification which is useful and simple to be applied by clinicians for triage and case management according to disease severity even in Primary Care settings and by epidemiologists working on disease surveillance; it reflects the natural course from mild to severe disease and covers all clinical manifestations.

Unfortunately, it is difficult to develop a single system which performs universally well under different circumstances, including pathogenesis studies or endpoint measures for vaccine or drug trials. Each of them has specific requirements. In our case, the model dengue +/− warning signs and severe dengue has been developed according to the needs for clinical management, especially triage and identification of severe cases which require special attention and those moving towards severe disease (warning signs). Furthermore, surveillance data can easier be standardized, addressing a long-standing problem of different case classifications used in different countries, incomparable data sets, and under-estimation of dengue burden. On the other hand, endpoint measurements of vaccine trials and studies of antiviral and/or disease modifying agents, as well as research on dengue pathogenesis may potentially benefit from the classification into levels of disease severity, but it has yet to be tested if for these purposes the revised classification designed for the clinician and for surveillance performs better than the DHF category.

First positive independent studies have been published, for example in Indonesia^16^ and Nicaragua,^17^ comparing the revised dengue case classification to DF/DHF/DSS with a prospective clinical dataset, concluding that the ‘revised dengue classification system may be better at detecting severe dengue infection cases compared to the WHO classification system’.

However, there has been a different views regarding the revised case classification,^18^,^19^ especially claiming that the DF/DHF/DSS classification was able to correctly identify cases of plasma leakage, as a predictor of severity and being related to the main underlying model of pathogenesis. But the same authors concluded in a separate study^13^ that only 68% of cases in need of interventions were classified as DHF with the DF/DHF/DSS classification criteria; therefore, 32% of severe cases would be missed. Dengue is nowadays a global disease, affecting all age groups, with multiple exposures to different viruses, often with co-morbidities and presenting with unusual manifestations; plasma leakage should be emphasized and not haemorrhage.

Furthermore, the use of haematocrit measurement is mentioned as a pre-requisite for assessing warning signs. However, one of the assumptions in the development of a revised dengue case classification was that diagnosis and treatment need to be often done without laboratory.

Also, the analysis of the shortcomings in the application of treatment guidelines (Steps 2 and 6) has shown that it is important to standardize clinical management, raise awareness about unnecessary interventions, match patient categories with specific treatment instructions, and make the key messages of patient management available to all health care staff dealing with dengue patients and that this is better achieved by applying the revised dengue classification with subsequent treatment guidelines. It is essential to work towards this aim of improved patient management in a harmonized way and the first steps have already been done.^9^

## Ethical Review

Ethical reviews have been obtained for each study according to the necessities of the study, e.g. for the prospective epidemiological cohort study with local and WHO ethical reviews, including consent forms of all cases. The same has been followed for all studies involving qualitative methods, e.g. interviews or focus groups with cases. This study, reviewing material in the public domain, does not require ethical review.

## Conflict of Interests

None of the authors have conflicts of interests and the necessary documentation has been submitted to the journal.

## Authorship Statement

OH is the main writer of the manuscript with substantial contributions to Steps 2–6 of the multi-step approach. AK is co-writer of the manuscript, with substantial contributions to all steps, including designing, developing, and implementing all steps. JF, LL, and EM contributed to the manuscript and have substantial contributions to Steps 2–7 (LL contributed also to Step 1). JLSM, JE, and RV contributed to the manuscript and contributed to several steps and to the overall process of implementing the revised case classification.

## Licence

The corresponding author has the right to grant on behalf of all authors and does grant on behalf of all authors, an exclusive licence (or non-exclusive for government employees) on a worldwide basis to Pathogens and Global Health.
